# Inhibitory Phenotype of HBV-Specific CD4^+^ T-Cells Is Characterized by High PD-1 Expression but Absent Coregulation of Multiple Inhibitory Molecules

**DOI:** 10.1371/journal.pone.0105703

**Published:** 2014-08-21

**Authors:** Bijan Raziorrouh, Malte Heeg, Peter Kurktschiev, Winfried Schraut, Reinhart Zachoval, Clemens Wendtner, Martin Wächtler, Michael Spannagl, Gerald Denk, Axel Ulsenheimer, Bertram Bengsch, Hanspeter Pircher, Helmut M. Diepolder, Norbert H. Grüner, Maria-Christina Jung

**Affiliations:** 1 Medical Department II and Institute for Immunology, University of LMU Munich, Munich, Germany; 2 Department of Nephrology/Rheumatology, University of Göttingen, Göttingen, Germany; 3 Leberzentrum, Munich, Germany; 4 Medical Department, Klinikum München-Schwabing, Munich, Germany; 5 Laboratory of Immunogenetics/Molecular Diagnostics, University of LMU Munich, Munich, Germany; 6 Medical Department II, University of Freiburg, Freiburg, Germany; 7 Department of Immunology, University of Freiburg, Freiburg, Germany; Hannover Medical School, Germany

## Abstract

**Background:**

T-cell exhaustion seems to play a critical role in CD8^+^ T-cell dysfunction during chronic viral infections. However, up to now little is known about the mechanisms underlying CD4^+^ T-cell dysfunction during chronic hepatitis B virus (CHB) infection and the role of inhibitory molecules such as programmed death 1 (PD-1) for CD4^+^ T-cell failure.

**Methods:**

The expression of multiple inhibitory molecules such as PD-1, CTLA-4, TIM-3, CD244, KLRG1 and markers defining the grade of T-cell differentiation as CCR7, CD45RA, CD57 and CD127 were analyzed on virus-specific CD4^+^ T-cells from peripheral blood using a newly established DRB1*01-restricted MHC class II Tetramer. Effects of *in vitro* PD-L1/2 blockade were defined by investigating changes in CD4^+^ T-cell proliferation and cytokine production.

**Results:**

CD4^+^ T-cell responses during chronic HBV infection was characterized by reduced Tetramer^+^CD4^+^ T-cell frequencies, effector memory phenotype, sustained PD-1 but low levels of CTLA-4, TIM-3, KLRG1 and CD244 expression. PD-1 blockade revealed individualized patterns of *in vitro* responsiveness with partly increased IFN-γ, IL-2 and TNF-α secretion as well as enhanced CD4^+^ T-cell expansion almost in treated patients with viral control.

**Conclusion:**

HBV-specific CD4^+^ T-cells are reliably detectable during different courses of HBV infection by MHC class II Tetramer technology. CD4^+^ T-cell dysfunction during chronic HBV is basically linked to strong PD-1 upregulation but absent coregulation of multiple inhibitory receptors. PD-L1/2 neutralization partly leads to enhanced CD4^+^ T-cell functionality with heterogeneous patterns of CD4^+^ T-cell rejunivation.

## Introduction

CD4^+^ T-cells are known to be critical components of virus-induced immune responses in terms of development, maintenance and control of T-cell and B-cell immunity. Detailed properties of CD4^+^ T-cell immunity during chronic viral infections remain to be defined in contrast to CD8^+^ T-cell responses. So far, virus-specific CD8^+^ T-cells during persisting viral diseases as human immunodeficiency virus (HIV), chronic hepatitis C virus (HCV) and chronic hepatitis B virus (CHB) infection become stepwise less functional and exhausted, a state characterized by hierarchical disruption of CD8^+^ T-cells to proliferate and to produce antiviral cytokines while memory T-cells perform vigorous effector functions [1]. Sustained coexpression of multiple inhibitory molecules such as programmed death-1 (PD-1), cytotoxic T lymphocyte-associated antigen-4 (CTLA-4), T-cell immunoglobulin domain and mucin domain 3 (TIM-3), CD244 (2B4) and killer cell lectin-like receptor G1 (KLRG1) were determined as common features strongly associated with CD8^+^ T-cell exhaustion. [2]–[7]. Functional data even indicated, that neutralization of these inhibitory pathways would be able to revive dysfunctional virus-specific CD8^+^ T-cells characterized by improvement of T-cell proliferation, cytotoxicity and cytokine production [3], [4], [7]–[9]. Indeed, while the role of inhibitory molecules in terms of CD8^+^ T-cell dysfunction is rather well characterized, a significant lack of data did exist with respect to the CD4^+^ T-cell compartment, although CD4^+^ T-cells are critical for successful viral control [10]. Recent data in chronic HIV and HCV infection revealed that high PD-1 expression seems to be associated with CD4^+^ T-cell dysfunction, with functional CD4^+^ T-cell rejuvenation following PD-L1/2 blockade [8], [11], [12]. Next to PD-1, sustained CTLA-4 expression in HIV infection demonstrated strong association with disease aggravation [7]. CD4^+^ T-cell dysfunction during HIV infection seems to be controlled by complex patterns of multiple coexpressed inhibitory receptors as previously described for CD8^+^ T-cells [2], [5], [9], [12]. However, the detailed role of PD-1, CTLA-4 and other inhibitory receptors as TIM-3, CD244 and KLRG1 on the development and maintenance of HBV-specific CD4^+^ T-cell dysfunction has yet to be elucidated. In this study, we therefore focused for the first time on the characterization of: ***(i)*** the memory and inhibitory phenotype of virus-specific CD4^+^ T-cells during chronic HBV infection by using a novel established DRB1*01-restricted MHC class II Tetramer and ***(ii)*** the functional impact of negative regulatory molecules as PD-1 measured by changes in CD4^+^ T-cell proliferation as well as IFN-γ, interleukin (IL)-2 and tumor necrosis factor (TNF)-α production.

## Material and Methods

### Study subjects

Peripheral blood was obtained from study subjects after institutional review board approval from the Ethic Committee of LMU Munich. All patients gave written informed consent. The protocol and the procedures of the study were conducted in conformity with ethical guidelines of the Declaration of Helsinki. Overall, 66 patients with chronic HBV infection (CHB), 41 patients with acute HBV infection (AHB), 5 HBV resolvers (RHB) and 7 healthy individuals were included (Table 1). Participant's age ranges from 18 to 65 years. Number of cases used for immunological T-cell assays are listed in detail in Table 2. Performance of one or more T-cell assays in each study subject was executed according to individual cell numbers. Patients with chronic infection have been seropositive for HBsAg for more than 6 months, anti-HBc and seronegative for HBs antibodies. Successful antiviral treatment with nucleotid/nucleosid analogs was defined as HBV DNA below 2.000 IU/ml. Patients with HCV, HDV and HIV co-infection were excluded. Acute HBV infection was diagnosed by the following criteria: acute onset of hepatitis in previously healthy individuals, along with recent onset of jaundice, exclusion of metabolic or toxic causes, ALT at least 10-fold upper the limit of normal, HBsAg and anti-HBc IgM positive. HBV resolvers are seronegative for HBsAg and seropositive for HBs antibodies.

**Table 1:** Baseline Characteristics*.

Hepatitis B virus infection	CHB^untreated^	CHB^treated^ **	AHB
number (n)	n = 42	n = 23	n = 8
sex (female/male)	20/22	7/16	2/6
age (years)	39 (19–65)	48 (18–65)	37 (24–63)
HBV DNA (IU/ml)	600	0	n.d.***
ALT (U/L)	36 (12–860)	33 (17–590)	400 (21–2100)
HBeAg (negative/positive/n.d.**)	28/6/8	10/5/8	0/2/6

*presented as median values.

**nucleosid/nucleotide analogs.

***not determined.

**Table 2:** Immunological assays and number of patient cases.

Material and Methods	CHB (n = 66*)	AHB (n = 41*)/RHB (n = 5)	healthy (n = 7*)
HLA-DRB1*01 positive	n = 66	n = 8/5	n = 7
Thymidine-based proliferation assay	n = 0	n = 38/5	n = 0
MHC class II Tetramer staining (HBV/EBV/Flu)	n = 30/15/21	n = 8/0/0/5/0/0	n = 7/0/0
CD4^+^ T-cell expansion assay	n = 23	n = 0/0	n = 0
ICS	n = 13	n = 0/0	n = 0

*overall number of patients included into study.

### Synthetic peptides

HLA-DRB1*01-restricted HBV core 61–80 (CWGELMTLATWVGVNLEDPA) and Influenza hemagglutinin (PKYVKQNTLKLAT) (purity: >90%) were used (EMC Microcollections).

### Proliferation assay

Thymidine-based proliferation assay was performed as described previously [13].

### Flow cytometric analysis

The following reagents were used: PD-1^FITC^, CTLA-4^APC^, CD4^FITC/APC-H7/V500^, CD14^PerCP^, CD19^PerCP^, CD3^PerCP^, Via Probe, Monensin, functional grade anti-CTLA-4 (BD Biosciences); CD244^FITC^, PD-1^APC^, CD127^APC-Cy7^, IL-2^FITC^, IFN-γ^PE^, TNF-α^PE-Cy7^, anti-PD-L1/2 and anti-IgG1 isotype control (eBioscience); TIM-3^APC^ (R&D Systems); CCR7^FITC^, CD57^PacificBlue^, CD45RA^PacificBlue^, functional grade anti-TIM-3 (Biolegend). Micro-Beads^anti-PE^ (Miltenyi). KLRG1^AlexaFluor488^ (clone: 13F12F2) was generated as described [14].

### MHC class II Tetramer staining

HLA-DRB1*01 MHC class II Tetramers (Beckman Coulter) were used as listed: HBV core 60-75^PE^ (LCWGELMTLATWVGVN), Influenza hemagglutinin^PE^ (PKYVKQNTLKLAT) and EBV EBNA1^PE^ (TSLYNLRRGTALA). Staining and calculation of Tetramer^+^ T-cells including enrichment step and CTLA-4 staining were performed as described [11] [15]. Briefly, lymphocytes were incubated for surface markers after staining for Tetramers. After wash step, cells were incubated with Micro Beads. 90% of cells were applied to MS Columns (Miltenyi). The other 10% were reserved for FACS (pre-enrichment sample). PE-positive cells were eluted from the column (post-enrichment sample) and analyzed by FACS. Tetramer frequencies were determined by dividing the number of Tetramer^+^CD4^+^ T-cells after enrichment by the total number of input CD4^+^ T-cells.

### *In vitro* expansion assay

*In vitro* expansion was performed with 2×10^6^ PBMC from chronically infected subjects. After pre-incubation with anti-PD-L1/2 (1 µg/2 µg/ml), anti-CTLA-4 (50 µg/ml) and anti-TIM-3 (10 µg/ml) for 30 min. at 37°C, cells were stimulated with HBV or Flu peptide (10 µg/ml). At day 7, 20 IU/ml recombinant human IL-2 (rhIL-2) was added. Based on CD4^+^ T-cell clone stimulation experiments Tetramer staining was performed at day 21 revealing highest expansion rate of virus-specific CD4^+^ T-cells.

### Intracellular Th-1 cytokine release (ICS)

After 21 days of *in vitro* expansion of 3×10^6^ PBMC with HBV core or Flu peptide in the presence or absence of anti-PD-L1/2 and 20 IU/ml rhIL-2, cells were restimulated with antigen (10 µg/ml) in the presence of autologous irradiated (4000 rads) PBMCs (1×10^5^/well) and Monensin (2 µM). After 6 hours of incubation, cells were stained with surface markers, fixed, permeabilized and stained with anti-IFN-γ, anti-IL-2 and anti-TNF-α. Cells were gated on CD3^+^CD4^+^ T-cells.

### Statistical Analysis

Data were shown as mean values with SEM (standard error of the mean). GraphPad Prism 6 was used for Mann-Whitney U test, Wilcoxon signed rank and Chi^2^-test. P values of less than 0.05 were considered significant. Significance of cytokine increase has been determined by calculating the difference between antigenic stimulation and PD-L1/2 blockade. Patients only exceeding pre-determined values (IFN-γ >0.38; IL-2 >0.07; TNF-α >0.2) were classified as cytokine responders upon PD-L1/2 blockade. Unspecific *in vitro* reaction was excluded by using matched isotype controls in T-cell expansion and ICS assays.

## Results

### Validation of DRB1*01-restricted MHC class II Tetramer

For phenotyping of HBV-specific CD4^+^ T-cells a DRB1*01-restricted MHC class II Tetramer was designed based on a previously characterized immunodominant epitope within the HBV core antigen region on amino acid position 61–80 [16]. We were able to demonstrate that epitope 61–80 was strongly recognized by CD4^+^ T-cells from DRB1*01-positive patients (Fig.S1A and B). CD4^+^ T-cell clone characterization demonstrated strong and specific Tetramer staining with a lower limit of detection of 0.001% of total CD4^+^ T-cells in titration experiments (Fig.S1C and D).

### Virus-specific CD4^+^ T-cell frequencies

**Figure 1:**
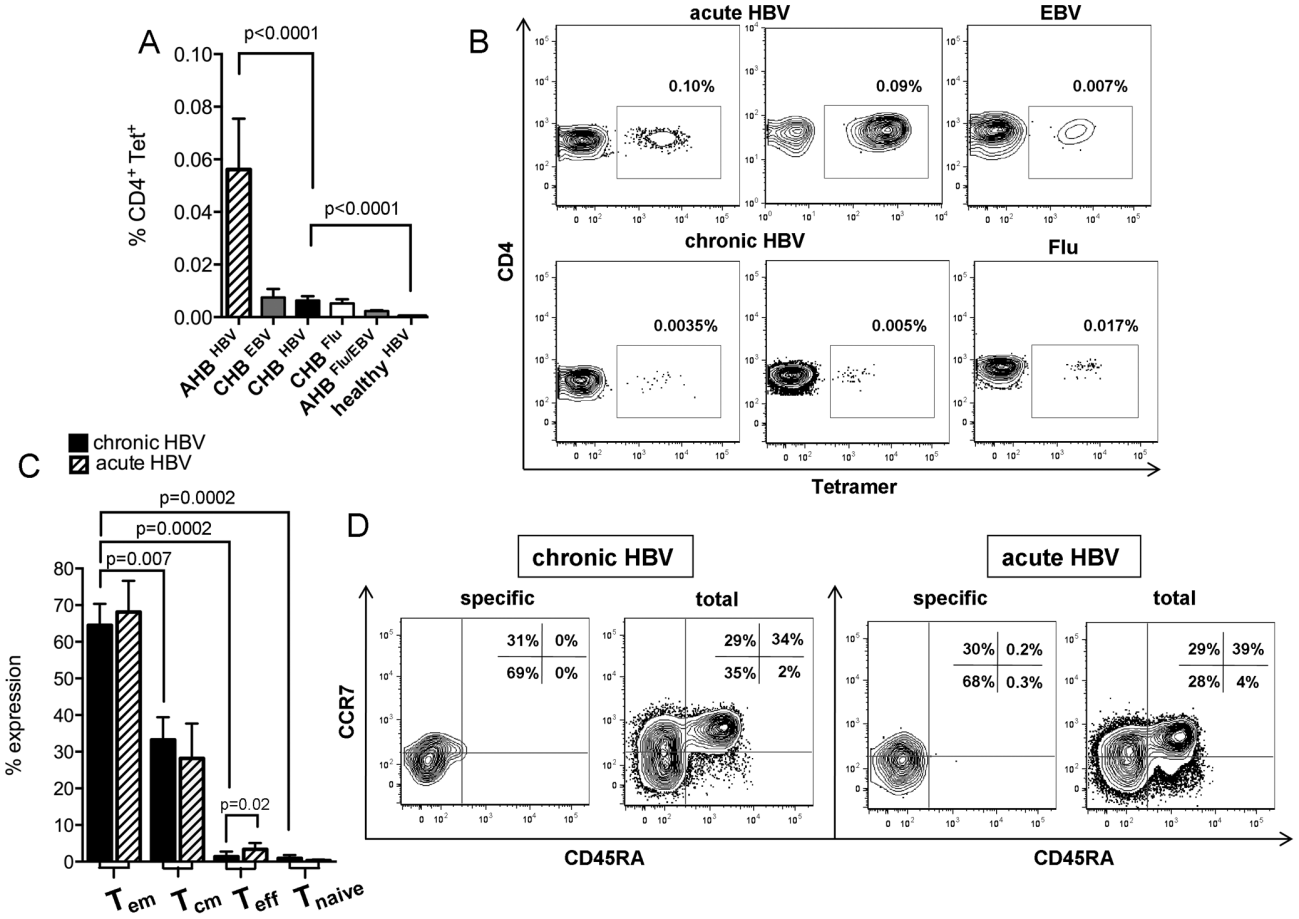
Virus-specific CD4^+^ T-cell frequencies and memory phenotype. (**A**) Tertamer^+^CD4^+^ T-cell frequencies in acutely (n = 8) and chronically (n = 30) infected HBV patients versus healthy controls (n = 7), influenza- and EBV-specific CD4^+^ T-cells as tested in CHB (Flu: n = 21; EBV: n = 15) and AHB (Flu/EBV: n = 6). (**B**) Representative contour plots are gated on CD14^−^, CD19^−^, Via Probe^−^ CD4^+^ T-cells following enrichment step. (**C**) Expression of CD45RA and CCR7 on Tetramer^+^CD4^+^ T-cells during chronic (*black bars*) (n = 8) and acute HBV (*striped bars*) (n = 6). (**D**) Contour plots of effector memory (T_em_; CCR7^neg^CD45RA^neg^), central memory (T_cm_; CCR7^pos^CD45RA^neg^), effector (T_eff_; CCR7^neg^CD45RA^pos^) and naive (T_naive_; CCR7^pos^CD45RA^pos^) CD4^+^ T-cell subsets during chronic and acute HBV.

First, we characterized virus-specific CD4^+^ T-cell frequencies during different courses of HBV infection. Study subjects with acute HBV displayed the highest frequencies (0.056%) as compared to chronic infection including untreated and treated patients (0.006%) (Fig.1A). We were able to detect Epstein-Barr-virus-specific and Influenza-specific CD4^+^ T-cells in CHB (EBV: 0.007%; Flu: 0.005%) and AHB (EBV: 0.0026%; Flu: 0.0016%) (Fig.1A). CD4^+^ T-cell frequencies in both viral entities as tested in acute and chronic HBV did not differ from frequencies in chronically infected HBV patients, but were declined compared to acute HBV infection (Fig.1A). Unspecific background of the HBV core Tetramer was analyzed by staining DRB1*01 positive healthy subjects (0.00%). Represantative stainings are shown in Figure 1B. In order to rule out variations of Tetramer frequencies with respect to viral load, we performed longitudinal measurements in 5 chronically infected patients before and during treatment with nucleosid/nucleotide analogs. Analysis revealed a slight decrease of CD4^+^ T-cell frequencies from the pre-treatment phase (0.015%) up to a 2-year follow-up with viral control upon therapy initiation (year 1: 0.0033%; year 2: 0.0015%) (data not shown). No significant differences in Tetramer frequencies exists regarding antiviral therapy and viral load, HBeAg status as well as GPT levels (data not shown).

### Virus-specific CD4^+^ T-cell differentiation

Successful immune control depends on balanced T-cell differentiation, why we analyzed in a first set of experiments the state of CD4^+^ T-cell differentiation during acute-resolving and chronic HBV infection according to the differentiation model of CD8^+^ T-cells [17]. Tetramer^+^CD4^+^ T-cells basically displayed the effector memory phenotype (Tem, CCR7^neg^CD45RA^neg^) in acute (68.1%) and chronic HBV (64.5%), followed by central memory CD4^+^ T-cells (Tcm, CCR7^pos^CD45RA^neg^) (Fig.1C). Terminally differentiated (CCR7^neg^CD45RA^pos^) and naive (CCR7^pos^CD45RA^pos^) CD4^+^ T-cells were almost absent (Fig.1C). Tem CD4^+^ T-cells during chronic HBV were more frequent than Tcm, Teff and Tnaive CD4^+^ T-cells. Overall, patterns of HBV-specific CD4^+^ T-cell differentiation revealed no differences in acute and chronic HBV infection as well as in chronic untreated and treated HBV patients (data not shown). Interestingly, total CD4^+^ T-cells in chronic and acute HBV infection displayed shortened but assimilable data for Tem (36.8% vs. 29.7%), Tcm (28.9% vs. 31.2%) and naive CD4^+^ T-cells (30.7% vs. 35.7%), with low frequencies for terminally differentiated CD4^+^ T-cells (3.3% vs. 3.2%), if compared to HBV-specific Tetramer^+^CD4^+^ T-cells. Representative stainings are shown in Figure 1D. As we know, that CD57 represents senescent and terminally differentiated lymphocytes with disrupted T-cell proliferation, we examined CD57 expression in HBV, EBV and Flu infection. CD57 is rarely expressed in acute (20.4%) and chronic HBV (5.9%) as well as on EBV- (17.4%) and Flu-specific CD4^+^ T-cells (0.8%) (Fig.S2A and B).

### Expression patterns of multiple inhibitory molecules in chronic HBV

**Figure 2:**
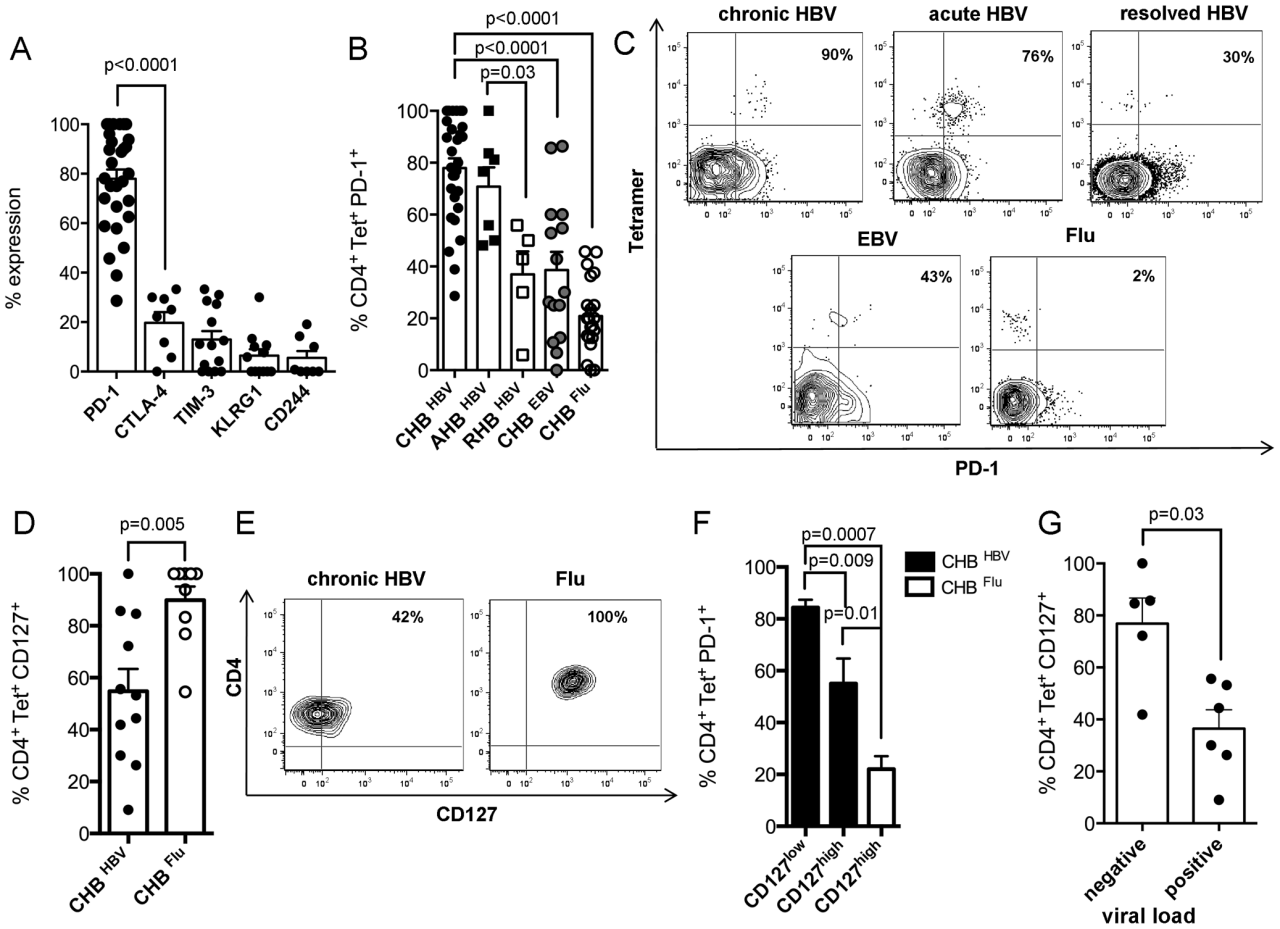
Inhibitory phenotype of HBV-specific CD4^+^ T-cells. (**A**) PD-1 (n = 29), CTLA-4 (n = 8), TIM-3 (n = 14), KLRG1 (n = 12) and CD244 (n = 8) expression is displayed on Tetramer^+^CD4^+^ T-cells during chronic HBV. (**B**) PD-1 expression in chronic (*black points*) (n = 29) and acute HBV (*black squares*) (n = 7), resolved HBV (*white squares*) (n = 5), EBV (*grey points*) (n = 15) and Flu (*white points*) (n = 21) infection. (**C**) Contour plots illustrate patterns of PD-1 expression during HBV, EBV and Flu infection. (**D**) CD127 expression on HBV-specific (*black points*) (n = 11) and Flu-specific CD4^+^ T-cells (*white points*) (n = 9). (**E**) Contour plots display CD127 expression on CD14^−^, CD19^−^, Via Probe^−^, Tetramer^+^ T-cells. (**F**) CD127 defines PD-1 expression as examined in HBV (*black bars*) and Flu infection (*white bar*). (**G**) Influence of viral load on CD127 expression.

Similar profiles of CD4^+^ T-cell differentiation in acute-resolving and chronic HBV infection suggested preserved ability of memory CD4^+^ T-cell generation even during persisting viral infection. We consecutively asked for further reasons for CD4^+^ T-cell dysfunction. Since we know that T-cell exhaustion is a common feature for dysfunctional CD8^+^ T-cells, we characterized multiple inhibitory molecules on virus-specific CD4^+^ T-cells of overall 30 chronically infected HBV patients. CD4^+^ T-cells most frequently expressed PD-1 (77.9%) in contrast to CTLA-4 (19.6%), TIM-3 (12.9%), KLRG1 (6.4%) and CD244 (5.5%) (Fig.2A). Chronic HBV infection was associated with strong PD-1 expression when compared to Flu-specific (20.9%) and EBV-specific CD4^+^ T-cells (38.6%) (Fig.2B). Patients with acute HBV displayed high PD-1 expression (70.7%), comparable to chronic HBV infection (Fig.2B). However, in case of viral clearance, PD-1 expression declined significantly in RHB (36.9%) (Fig.2B). To define the influence of T-cell differentiation on the inhibitory phenotype, we determined PD-1 on Tcm, Tem and naive CD4^+^ T-cells. PD-1 expression was quite similar expressed on Tcm (77%) and Tem (72.1%), but strongly decline on naive CD4^+^ T-cells (1.5%) (data not shown). Notably, virus-specific PD-1 expression in CHB was not influenced by antiviral therapy and viral load as dissected in Figure S2C. Stainings of PD-1 expression are shown in Figure 2C.

### CD127 defines virus-specific PD-1 expression

Next, we asked for the linkage of CD127 and PD-1 expression as shown for CD8^+^ T-cells [2], [18]. CD127 was downregulated during chronic HBV (54.8%) as compared to Flu infection (n = 9) (89.8%) (Fig.2D). Representative stainings are shown in Figure 2E. Depending on CD127 expression above or below 60% (pre-defined cut-off in healthy subjects), we defined CD127^high^ and CD127^low^ expressing CD4^+^ T-cells. Tetramer^+^CD127^high^CD4^+^ T-cells displayed less PD-1 expression (55% vs. 84.3%) (Fig.2F) and decreased viral load (10 vs. 1.7×10^6^ IU/ml) (Fig.2G) as compared to CD127^low^CD4^+^ T-cells (p = 0.009 and p = 0.02, respectively).

### PD-L1/2 blockade reactivates CD4^+^ T-cell proliferation

**Figure 3:**
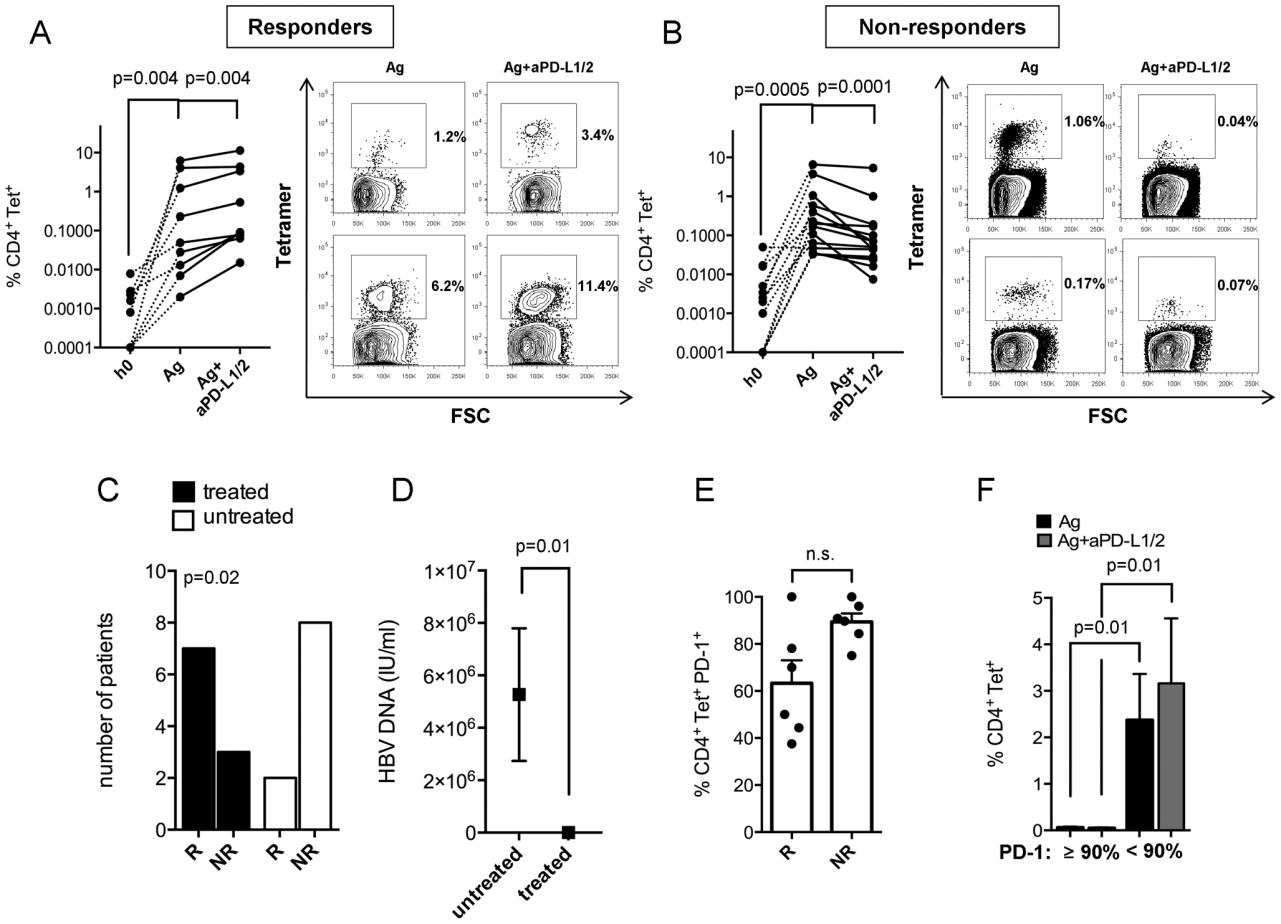
Effect of PD-L1/2 blockade on CD4^+^ T-cell expansion. Induction of CD4^+^ T-cell proliferation in chronic HBV (n = 23) from h0 (*left*) to antigenic stimulation (*middle*) and PD-L1/2 blockade (*right*) illustrated as point to point graphs from (**A**) PD-L1/2 responders (n = 9) and (**B**) Non-responders (n = 14). Contour plots are shown for each group. (**C**) Patients treated with nucleosid/nucleotid analogs (*black bars*) responded to PD-1 blockade using Chi^2^-test, indicating that viral control let enhance patients susceptibility as (**D**) treated patients are characterized by low viremia. (**E**) Decreased PD-1 expression in PD-L1/2 responders (R) (n = 6) versus non-responders (NR) (n = 6). (**F**) CD4^+^ T-cell frequencies upon antigenic stimulation (*black bars*) and PD-L1/2 inhibition (*grey bars*) in relation to PD-1 expression.

Further, we characterized the reactivation of CD4^+^ T-cell proliferation during chronic HBV following PD-L1/2 neutralization in the presence of HBV core antigen as PD-1 represents the strongest linkage to chronic HBV. We were able to classify: ***(1)*** PD-L1/2 responders (R) with enhancement of Tetramer^+^CD4^+^ T-cell frequencies from 1.3% to 2.2% (p = 0.004) (Fig.3A) and ***(2)*** PD-L1/2 non-responders (NR) with decreased frequencies from 0.9% to 0.5% (p = 0.0001) (Fig.3B). Both groups revealed strong CD4^+^ T-cell expansion from *ex vivo* frequencies of 0.002% (R) and 0.007% (NR) to 1.3% (R) and 0.9% (NR) upon antigenic restimulation. Notably, treated patients with long-term viral control commonly responded to PD-1 blockade, while untreated high viremic (5.2×10^6^ IU/ml) subjects failed to revive T-cell proliferation (p = 0.02 and p = 0.01, respectively) (Fig.3C and D). Interestingly, responsiveness of PD-1 neutralization was not associated with viral load within the group of chronically untreated HBV patients (data not shown). PD-L1/2 responders are characterized by slightly reduced PD-1 expression (63.3%) in contrast to non-responders (89.3%) (Fig.3E). Indeed, CD4^+^ T-cell responsiveness seems to depend on PD-1 expression (Fig.3F). We found, that PD-1 expression higher than 90% (PD-1^high^) versus PD-1 less than 90% (PD-1^low^) limited the ability of CD4^+^ T-cells to proliferate in response to antigen (p = 0.01) and PD-L1/2 blockade (p = 0.01). Of note, PD-L1/2 inhibition failed to rescue Flu-specific CD4^+^ T-cell proliferation (Fig.S3). Next, we asked for the effect of CTLA-4 and TIM-3 inhibition on CD4^+^ T-cell expansion. Neither CTLA-4 (p = 0.21) nor TIM-3 (p = 0.12) blockade were able to reactivate Tetramer^+^CD4^+^ T-cell frequencies in chronically infected HBV patients when compared to antigenic stimulation alone (Fig.S3).

### PD-L1/2 blockade improves the ability of CD4^+^ T-cells to produce Th-1 cytokines

**Figure 4:**
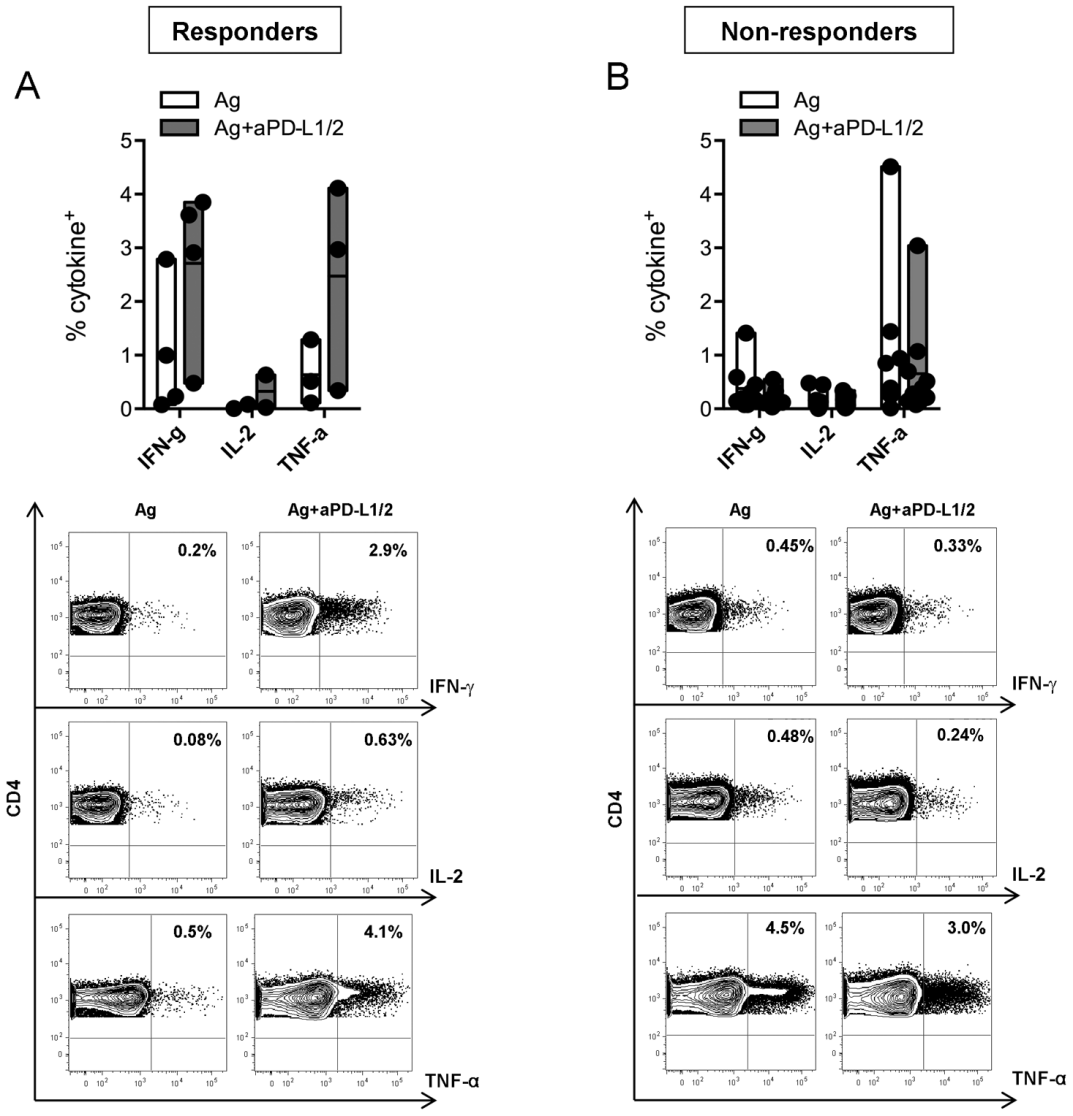
Effect of PD-L1/2 blockade on cytokine release. Analysis of cytokine^+^CD4^+^ T-cells allows to differ (**A**) PD-L1/2 responders (n = 4) from (**B**) Non-responders (n = 9). Floating bars illustrate mean values ranging from minimun to maximum production of IFN-γ, IL-2 and TNF-α seperatly following antigen stimulation (*white bars*) and PD-1 blockade (*grey bars*). Contour plots are shown for IFN-γ (*top*), IL-2 (*middl*e) and TNF-α (*bottom*) secretion upon antigen stimulation (*left*) and PD-1 pathway neutralization (*right*) after gating on CD3^+^CD4^+^ T-cells.

Next to T-cell proliferation, we addressed the question if PD-1 inhibition would be able to reactivate IFN-γ, IL-2 and TNF-α production. PD-L1/2 blockade reactivates at least one of the investigated cytokines in 4 of 13 chronic HBV patients, classified as PD-L1/2 responders with enhanced cumulative CD4^+^ T-cell-related cytokine production from 0.67% to 2.1% (p = 0.004). Cytokine rejunivation upon PD-L1/2 inhibition was almost characterized by increased CD4^+^IFN-γ^+^ (1.0% to 2.7%) and CD4^+^TNF-α^+^ T-cells (0.63% to 2.47%) with only slight enhancement of CD4^+^IL-2^+^ T-cells (0.046% to 0.32%) (Fig.4A). In contrast, 9 of 13 patients were classified as PD-L1/2 non-responders with an overall decrease of cytokine production from 0.47% to 0.31% (p = 0.002). Declined patterns of CD4^+^IFN-γ^+^ (0.38% to 0.19%), CD4^+^IL-2^+^ (0.14% to 0.096%) and CD4^+^TNF-α^+^ (0.93% to 0.65%) are dissected in Figure 4B.

## Discussion

Upregulation of inhibitory molecules during chronic viral infections display a critical mechanism thought to be crucial for CD8^+^ T-cell failure [2]–[5], [7], [19]. Sustained PD-1 and CTLA-4 expression was highlighted as a hallmark for CD8^+^ T-cell exhaustion with rejunivation of T-cell function following *in vitro* blockade of both molecules [20], [21]. CD8^+^ T-cell exhaustion is additionally associated with coexpression of PD-1, CTLA-4, TIM-3 and CD244 with improvement of T-cell responses following simultaneous neutralization [2], [7], [9], [19], [22]. In contrast, CD4^+^ T-cell exhaustion remains less well understood, although CD4^+^ T-cells are critical for the control and elimination of viral diseases [10]. A major limitation for virus-specific CD4^+^ T-cell characterization is their relative paucity once chronic infection is established [23]-[25]. To illuminate features of CD4^+^ T-cell dysfunction during chronic HBV infection, we analyzed ***(i)*** the memory and inhibitory phenotype of Tetramer^+^CD4^+^ T-cells as well as ***(ii)*** the capacity of PD-L1/2 neutralization to reactivate CD4^+^ T-cell function as PD-1 expression is suggested to be crucial during chronic HBV. Although MHC class II Tetramers are rarely used for analysis of antigen-specific CD4^+^ T-cells, we established one newly DRB1*01-restricted Tetramer to track antigen-specific CD4^+^ T-cells during HBV infection [10], [11], [15], [25]. Our results indicate, that Tetramer^+^CD4^+^ T-cells are reliably detectable even during the chronic phase of HBV assigning one basic principle for CD4^+^ T-cell phenotyping. Of note, it must be pointed out that results are in reference to CD4^+^ T-cells only specific to HBV core epitope 61–80. As we know, that immune control depends on balanced distribution of effector and memory T-cells, we first determined the state of virus-specific CD4^+^ T-cell differentiation in acute-resolving and chronic HBV infection. CD4^+^ T-cells predominantly displayed the effector memory phenotype as described for HCV and LCMV infection, indicating that CD4^+^ T-cells during chronic HBV have the capacity to transit through an effector stage with faultless first line defence as acute-resolving patients express comparable frequencies of Tem CD4^+^ T-cells. [26], [27]. Since we know that CD8^+^ T-cell exhaustion is associated with sustained expression of multiple inhibitory molecules, we defined the impact of a set of inhibitory receptors and were able to demonstrate that exclusively PD-1 correlated to chronic HBV infection. Surprisingly, CD4^+^ T-cells even displayed reduced CTLA-4, TIM-3, KLRG1 and CD244 expression as compared to CD8^+^ T-cells, suggesting less exhausted CD4^+^ T-cells [2]–[5], [19]. In the face of depressed expression of inhibitory molecules, we consecutively asked for T-cell senescence as a possible pathomechanism next to T-cell exhaustion resulting in CD4^+^ T-cell dysfunction. However, low frequencies of CD57 expression propably implicate less significance of CD4^+^ T-cell senescence in chronic HBV infection. PD-1 expression in chronic HBV infection was not influenced by viral load as previously shown within other viral entities [7], [11], [28]. Elevated PD-1 expression as described in acute HBV infection might play a mechanistic role in the prevention of overwhelming CD4^+^ T-cell immunity to avoid T-cell-mediated acute liver failure. Importantly, PD-1 expression seemed to be inversely correlated to CD127 as described for CD8^+^ T-cells [2], [18]. Low PD-1 expression came along with high CD127 expression, indicating that high PD-1 expression led to a diminished capability of CD4^+^ T-cells to maintain memory potential, although similar profiles of T-cell differentiation are shown for acute-resolving and chronic HBV. Indeed, higher PD-1 expression was associated with disrupted CD4^+^ T-cell expansion, while CD4^+^ T-cells with PD-1 expression less than 90% regain their proliferative capacity. Functional experiments testing the effect of PD-L1/2 neutralization on CD4^+^ T-cell expansion identified individualized patterns of responsiveness with enhanced T-cells proliferation almost in treated patients with successful viral control. These data are novel and provide important findings to immunological approaches for prospective therapeutic interventions in persisting HBV, but have limited validity due to low numbers of patients investigated. Importantly, our data even suggest that CTLA-4 and TIM-3 single blockade failed to reactivate CD4^+^ T-cell function, which is limited by low numbers of patients investigated but probably denoted a fundamental insusceptibility of HBV-specific CD4^+^ T-cells as previously described for CD8^+^ T-cells in chronic HCV infection [9]. In contrast, Nebbia et al. was able to rescue HBV-specific CD8^+^ T-cell responses by blocking TIM-3/galectin-9 interactions complementary to PD-1 pathway inhibition, suggesting distinct suppressive effects of inhibitory molecules as TIM-3 on CD4^+^ and CD8^+^ T-cells [22]. Associations of reduced PD-1 expression with improved PD-L1/2 responsiveness are consistent with previous reports in HCV infection [6], [11], [29]. In contrast, PD-1^high^CD4^+^ T-cells in HIV are more susceptible to PD-L1 blockade than PD-1^low^CD4^+^ T-cells, suggesting that functional influences of PD-1 might differ between viral entities [8]. In our experiments, PD-1 blockade partially improves the ability of CD4^+^ T-cells to produce IFN-γ, IL-2 and TNF-α. This entire reduced reactivation of cytokine release was still described in HCV and emphasizes limited ability of PD-L1/2 neutralization to revitalize CD4^+^ T-cell effector functions [11]. Described heterogeneous patterns of CD4^+^ T-cell reactivation might be explained by ***(i)*** the complex interaction of inhibitory molecules, ***(ii)*** the impact of different transcriptional molecules as T-bet and EOMES [30], [31], and ***(iii)*** the altered expression of T-cell receptor-mediated downstream signalling molecules. Further studies illuminating these questions particularly regarding clinical parameters would be necessary to provide additional insights into patterns of CD4^+^ T-cell reversibility in HBV. In summary, we characterized for the first time the disrupted CD4^+^ T-cell immunity during chronic HBV infection by defining the inhibitory phenotype and the changes of CD4^+^ T-cell function upon PD-1 inhibition using a novel MHC class II Tetramer. These findings provide new insights into the mechanisms underlying CD4^+^ T-cell dysfunction and revealed that exhausted HBV-specific CD4^+^ T-cells were strongly linked to high PD-1 expression with reactivation of CD4^+^ T-cell proliferation which depends on treatment-associated viral control.

## Supporting Information

Figure S1 **Validation of MHC class II Tetramer.** (**A**) HBV core antigens 51–70, 61–80 and 111–130 yielded the strongest response in acute HBV (n = 38) as 3 of 18 overlapping peptides (20 mer) covering the core region using 3H-thymidine proliferation assay. (**B**) Epitope 61–80 was most frequently recognized by DRB1*01-positive patients (n = 4) with acute HBV. (**C**) Staining of clone cells specific for epitope 60–75 with unspecific HCV Tetramer 1806–1818 (*left*) and specific HBV Tetramer 60–75 (*right*). (**D**) Clone cells were titrated into Tetramer negative PBMC. The number of added clone cells was correlated to the number of cells detected by specific Tetramer.
(TIF)

Figure S2 **T-cell senescence and PD-1 expression in correlation to viral load.** (**A**) CD57 revealed low expression in acute HBV (n = 6), EBV (n = 6), chronic HBV (n = 12) and Flu (n = 9) infection, indicating less significance of T-cell senescence during AHB and CHB. (**B**) Contour plots display virus-specific CD57 expression after gating on CD14^−^, CD19^−^, Via Probe^−^ CD4^+^ T-cells. (**C**) Bar graphs from chronic untreated (*black bars*) >2.000 IU/ml (n = 5), <2.000 IU/ml (n = 12) and treated (*white bar*) (n = 8) HBV patients are illustrating no differences in virus-specific PD-1 expression in correlation to viral load.
(TIF)

Figure S3 **Effect of PD-L1/2 blockade on Flu-specific as well as CTLA-4 and TIM-3 blockade on HBV-specific CD4^+^ T-cell expansion.** (**A**) Induction of Flu-specific CD4^+^ T-cell proliferation in chronically infected HBV patients (n = 8) from h0 (*left*) to antigenic re-stimulation (*middle*) and PD-L1/2 blockade (*right*) illustrated as point to point graphs. Contour plots are shown for Tetramer^+^CD4^+^ T-cell proliferation upon antigen stimulation (*left*) and PD-1 neutralization (*right*). (**B**) Induction of CD4^+^ T-cell proliferation in chronically infected HBV patients following CTLA-4 (*left*) (n = 7) and TIM-3 (*right*) (n = 4) blockade illustrated as point to point graphs.
(TIF)
