# Supplementary figures and images for: Inhibitory Phenotype of HBV-Specific CD4+ T-Cells Is Characterized by High PD-1 Expression but Absent Coregulation of Multiple Inhibitory Molecules
Source: PLoS One. 2014 Aug 21;9(8):e105703. doi: 10.1371/journal.pone.0105703 (PMC4140833; doi:10.1371/journal.pone.0105703)

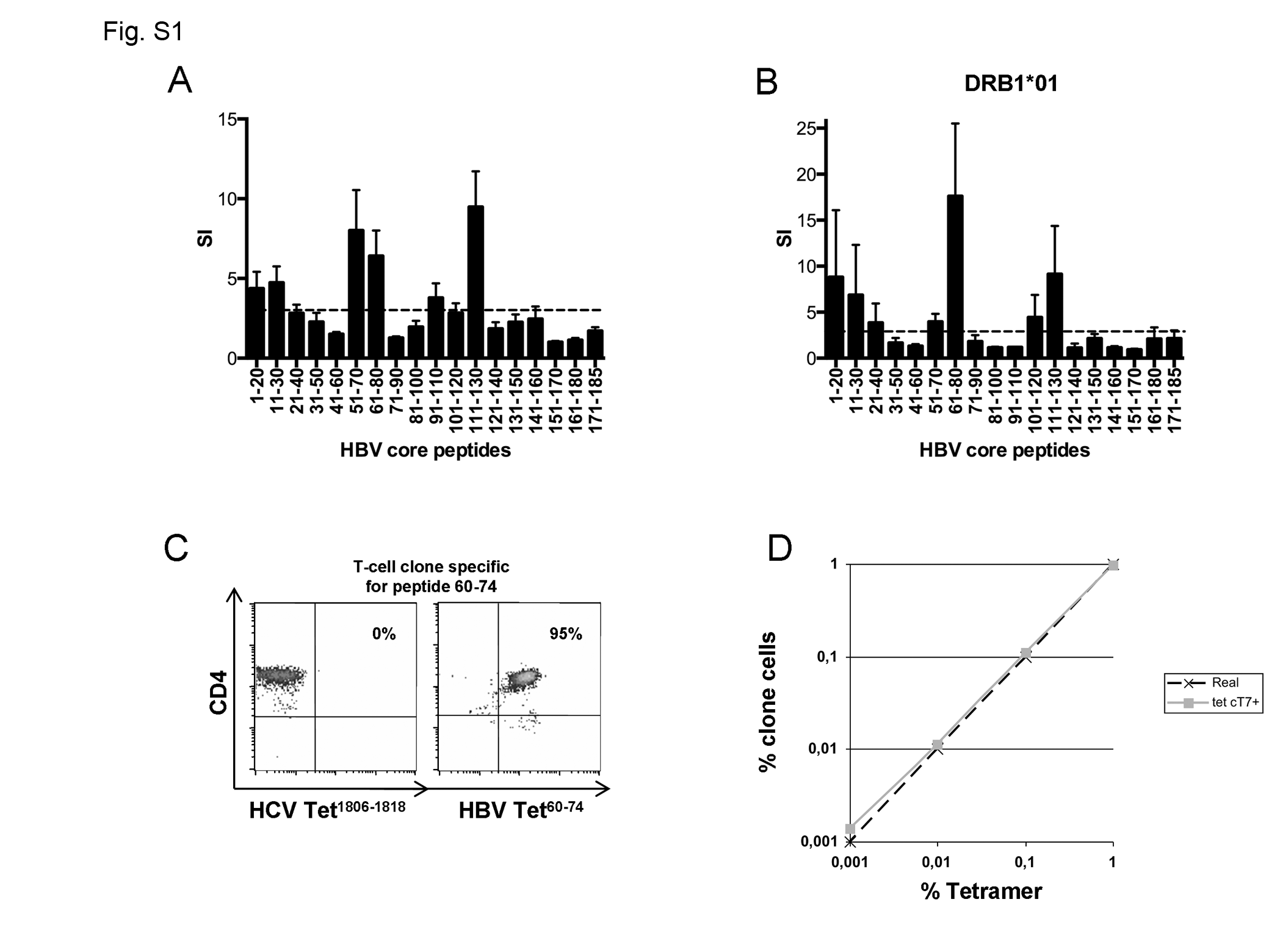

Supplement: Figure S1 — Validation of MHC class II Tetramer. (A) HBV core antigens 51–70, 61–80 and 111–130 yielded the strongest response in acute HBV (n = 38) as 3 of 18 overlapping peptides (20 mer) covering the core region using 3H-thymidine proliferation assay. (B) Epitope 61–80 was most frequently recognized by DRB1*01-positive patients (n = 4) with acute HBV. (C) Staining of clone cells specific for epitope 60–75 with unspecific HCV Tetramer 1806–1818 (left) and specific HBV Tetramer 60–75 (right). (D) Clone cells were titrated into Tetramer negative PBMC. The number of added clone cells was correlated to the number of cells detected by specific Tetramer. (TIF) [file pone.0105703.s001.tif]

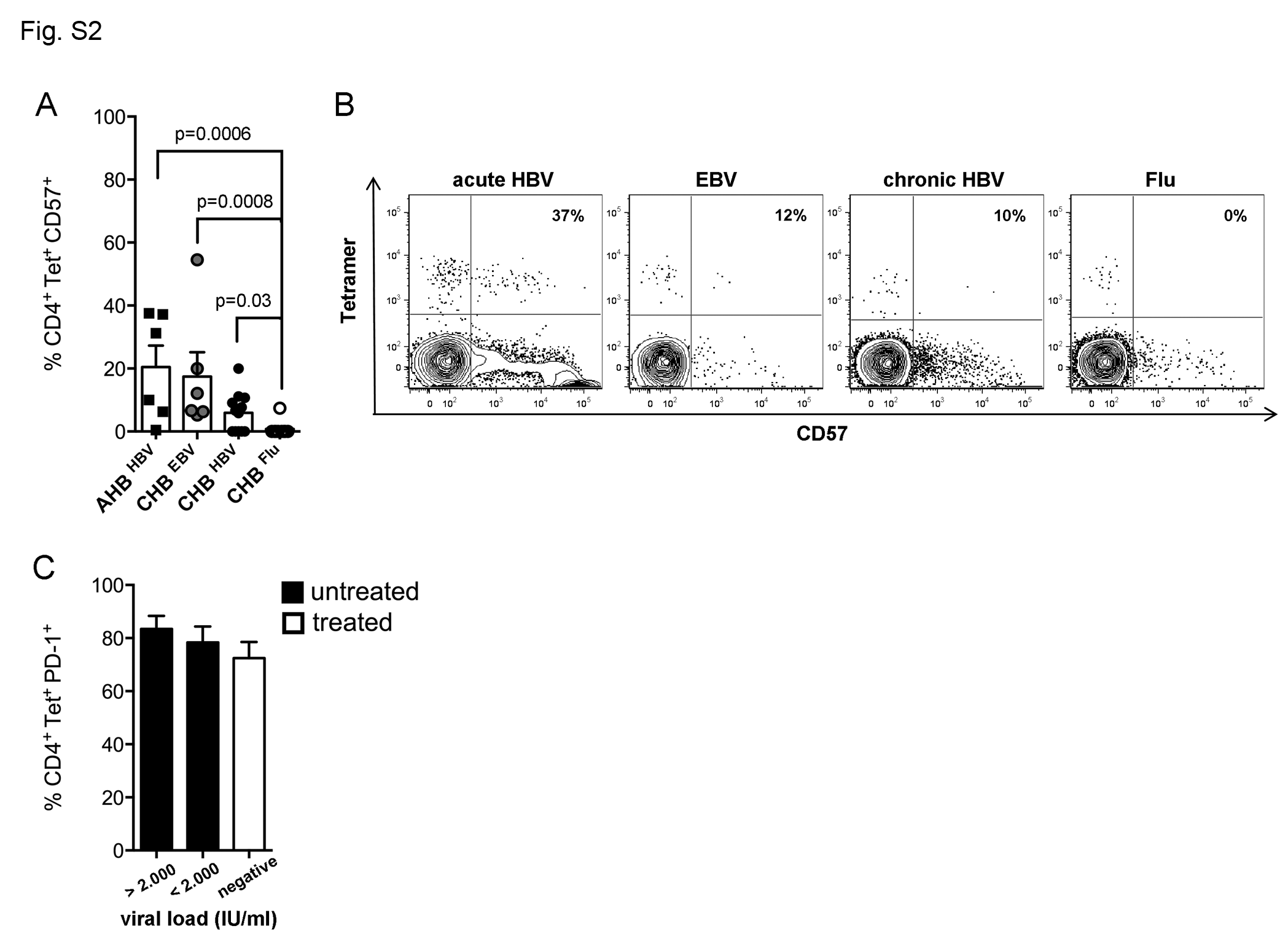

Supplement: Figure S2 — T-cell senescence and PD-1 expression in correlation to viral load. (A) CD57 revealed low expression in acute HBV (n = 6), EBV (n = 6), chronic HBV (n = 12) and Flu (n = 9) infection, indicating less significance of T-cell senescence during AHB and CHB. (B) Contour plots display virus-specific CD57 expression after gating on CD14−, CD19−, Via Probe− CD4+ T-cells. (C) Bar graphs from chronic untreated (black bars) >2.000 IU/ml (n = 5), <2.000 IU/ml (n = 12) and treated (white bar) (n = 8) HBV patients are illustrating no differences in virus-specific PD-1 expression in correlation to viral load. (TIF) [file pone.0105703.s002.tif]

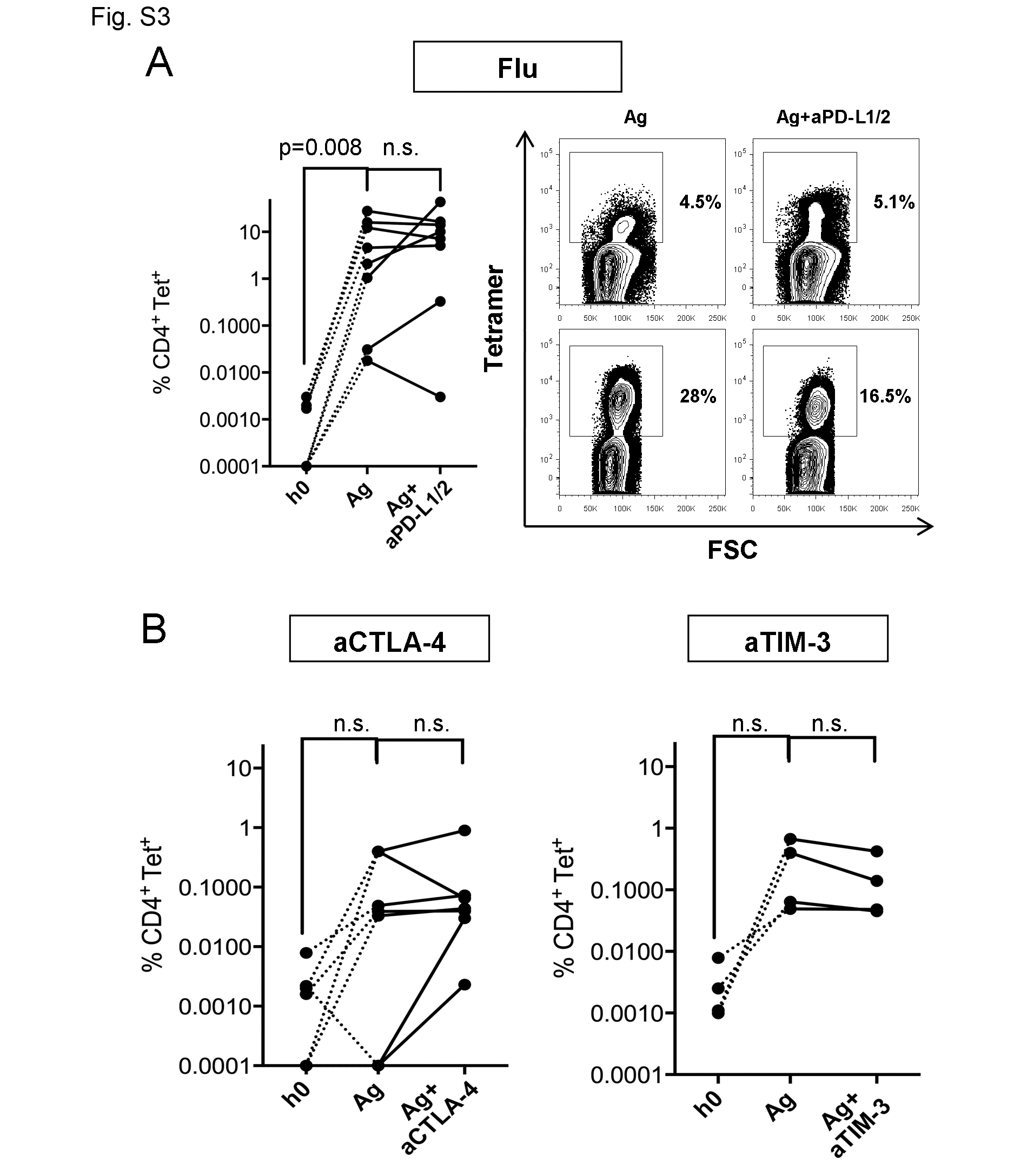

Supplement: Figure S3 — Effect of PD-L1/2 blockade on Flu-specific as well as CTLA-4 and TIM-3 blockade on HBV-specific CD4+ T-cell expansion. (A) Induction of Flu-specific CD4+ T-cell proliferation in chronically infected HBV patients (n = 8) from h0 (left) to antigenic re-stimulation (middle) and PD-L1/2 blockade (right) illustrated as point to point graphs. Contour plots are shown for Tetramer+CD4+ T-cell proliferation upon antigen stimulation (left) and PD-1 neutralization (right). (B) Induction of CD4+ T-cell proliferation in chronically infected HBV patients following CTLA-4 (left) (n = 7) and TIM-3 (right) (n = 4) blockade illustrated as point to point graphs. (TIF) [file pone.0105703.s003.tif]
